# Baseline and interim [18F]FDG-PET/MRI to assess treatment response and survival in patients with M0 esophageal squamous cell carcinoma treated by curative-intent therapy

**DOI:** 10.1186/s40644-023-00630-2

**Published:** 2023-11-06

**Authors:** Yin-Kai Chao, Chun-Bi Chang, Yu-Chuan Chang, Sheng-Chieh Chan, Chien-Hung Chiu, Shu-Hang Ng, Jason Chia-Hsun Hsieh, Jen-Hung Wang

**Affiliations:** 1 Division of Thoracic Surgery, Chang Gung Memorial Hospital-Linko, Chang Gung University, Taoyuan, 333423 Taiwan; 2 Department of Diagnostic Radiology, Linkou Chang Gung Memorial Hospital, Chang Gung University, Taoyuan, 333423 Taiwan; 3 Department of Nuclear Medicine and Molecular Imaging Center, Chang Gung Memorial Hospital-Linko, Chang Gung University, Taoyuan, 333423 Taiwan; 4 Department of Nuclear Medicine, Hualien Tzu Chi Hospital, Buddhist Tzu Chi Medical Foundation, Hualien, 970423 Taiwan; 5 School of Medicine, Tzu Chi University, Hualien, 970423 Taiwan; 6 Division of Hematology/Oncology, Department of Internal Medicine, Linkou Chang Gung Memorial Hospital, Chang Gung University, Taoyuan, 333423 Taiwan; 7 Department of Medical Research, Hualien Tzu Chi Hospital, Buddhist Tzu Chi Medical Foundation, Hualien, 970423 Taiwan

**Keywords:** Esophageal cancer, Positron-emission tomography, MRI, PET/MRI, Prognosis

## Abstract

**Background:**

To investigate the value of [18F]FDG-PET/MRI in predicting treatment response and survival in patients with primary M0 esophageal squamous cell carcinoma.

**Methods:**

Patients with esophageal squamous cell carcinoma received [18F]FDG-PET/MRI at baseline and during neoadjuvant or definitive chemoradiotherapy. The treatment response was classified according to the Response Evaluation Criteria for Solid Tumors 1.1. We used Kaplan-Meier and Cox regression analyses to assess the association between PET/MRI parameters and overall survival (OS) or progression-free survival (PFS).

**Results:**

We included 40 M0 patients in the final analysis. The volume transfer constant (*K*^*trans*^) from baseline PET/MRI (area under the curve (AUC) = 0.688, *P* = 0.034) and total lesion glycolysis (TLG) from baseline PET/MRI (AUC = 0.723, *P* = 0.006) or interim PET/MRI (AUC = 0.853, *P* < 0.001) showed acceptable AUC for predicting treatment response. The TLG from interim PET/MRI (interim TLG, *P* < 0.001) and extracellular volume fraction (*Ve*) on interim PET/MRI (interim *Ve*, *P* = 0.001) were identified as independent prognostic factors for OS. Baseline *Ve* (*P* = 0.044) and interim TLG (*P* = 0.004) were significant predictors of PFS. The c-indices of the prognostic models combining interim TLG with *Ve* for predicting OS, and baseline *Ve* and interim TLG for predicting PFS were 0.784 and 0.699, respectively. These values were significantly higher than the corresponding c-indices of the TNM staging system (*P* = 0.002 and *P* = 0.047, respectively).

**Conclusions:**

Combining the baseline and interim [18F]FDG-PET/MRI qualitative imaging parameters aids in predicting the prognosis of patients with M0 esophageal squamous cell carcinoma.

**Trial registration:**

The study was registered at Clinicaltrials.gov (identifier: NCT 05855291 and NCT 05855278).

**Supplementary Information:**

The online version contains supplementary material available at 10.1186/s40644-023-00630-2.

## Background

Esophageal cancer is ranked eighth in terms of cancer incidence and sixth in terms of cancer-related mortality [1]. The primary histological subtypes esophageal squamous cell carcinoma (ESCC) and esophageal adenocarcinoma represent two distinct entities with different epidemiological distributions, risk factors, and prognoses [2]. Esophageal squamous cell carcinoma is characterised by a poor prognosis, with a 5-year survival rate of 10-30% in most countries [3].

Patients with locoregionally advanced ESCC are generally treated with either definitive chemoradiotherapy (CRT) or neoadjuvant CRT, followed by surgery [4, 5]. However, patients who do not exhibit a positive response to CRT may face challenges due to the time delay caused by an ineffective therapeutic intervention. Therefore, it is essential to find a diagnostic test for the early prediction of treatment response to changes in the treatment regimen in non-responders. Unfortunately, a reliable biomarker for predicting treatment response in ESCC is currently unavailable. The assessment of therapeutic response through volume reduction in anatomic imaging, such as computed tomography (CT), is not highly accurate due to the delay of several weeks to months in tumor shrinkage after treatment. The reported sensitivity and specificity of CT in evaluating the response after CRT range from 33 to 55% and 50–71%, respectively [6].

Studies attempting to optimize treatment strategies using novel imaging modalities have focused on the potential role of [18F]FDG-PET in patients with ESCC. [18F]FDG-PET typically demonstrates a rapid reduction in tumor signals after effective treatment, antedating decrease in tumor size. In a meta-analysis, Cong et al. reported that [18F]FDG-PET showed a pooled sensitivity and specificity of 67% and 69%, respectively, in assessing the treatment response after neoadjuvant CRT in patients with ESCC [7]. [18F]FDG-PET performed during CRT (interim PET) showed pooled sensitivity and specificity of 85% and 59%, respectively. The predictive power of [18F]FDG-PET alone remains suboptimal for predicting the treatment response in patients with ESCC treated with CRT.

Functional MRI visualizes different and possibly complementary tumor characteristics to glycolysis on [18F]FDG-PET. Diffusion-weighted MR imaging (DWI) quantifies the diffusion motion of water molecules, and the apparent diffusion coefficient (ADC) derived from DWI demonstrated prognostic implications in patients with esophageal cancer [8]. Dynamic contrast-enhanced MRI (DCE-MRI) provides information on tissue perfusion and microcirculation. Heethuis et al. demonstrated that changes in DCE-MRI perfusion parameters during neoadjuvant CRT could predict histopathologic response in patients with esophageal cancer [9].

Hybrid PET/MRI systems enable the acquisition of anatomical, functional, and metabolic information during the same session. Various imaging biomarkers from [18F]FDG-PET, DCE-MRI, and DWI can be obtained in a single PET/MRI examination. However, there is a paucity of prospective data defining the utility of PET/MRI in predicting treatment response and survival in patients with ESCC. To address these concerns, we conducted a prospective study to investigate the prognostic value of [18F]FDG-PET/MRI in patients with primary ESCC after chemoradiotherapy.

## Methods

### Patients

This prospective trial evaluated the performance of baseline and interim [18F]FDG-PET/MRI in patients with primary esophageal squamous cell carcinoma undergoing definitive therapy. Patients with a histological diagnosis of primary esophageal squamous cell carcinoma scheduled to receive definitive chemoradiotherapy or neoadjuvant chemoradiotherapy followed by surgery were eligible. We excluded pregnant or lactating patients, patients who had M1 disease, or those with contraindications of MRI. The enrolled patients received both [18F]FDG-PET/CT and [18F]FDG-PET/MRI before treatment (baseline) and after receiving 20 Gy of radiotherapy (interim). Baseline scans were completed within the initial two weeks before the commencement of treatment. Interim PET scans were conducted approximately two weeks after the initiation of treatment. This study was approved by the Institutional Review Board of Chang Gung Memorial Hospital and conducted in accordance with the Declaration of Helsinki. All patients provided written informed consent before participating and could withdraw from the study at any time. This trial was registered at Clinicaltrials.gov (identifier NCT05855291 and NCT05855278).

### [18F]F-FDG-PET/MRI

[18F]FDG -PET/MRI was conducted following [18F]FDG-PET/CT on the same day. Before [18F]FDG-PET/CT imaging, the patients fasted for at least 6 h. The scan was performed with a Biograph mCT scanner. The emission images were acquired from the vertex to the mid-thigh region within 50 to 70 min following the administration of [18F]FDG (370 MBq). Each table position was scanned for 1.5 min. Following the completion of the PET/CT scan, the patient was transferred to the PET/MRI machine for the subsequent scan, with an average time lapse of 39 min between the two imaging sessions. Accordingly, the time interval for patients to undergo PET/MRI scanning was around 114 min after tracer administration. PET/MRI imaging was conducted using a Biograph mMR system manufactured by Siemens Healthcare in Erlangen, Germany. This scanner utilized A 3-T magnetic field strength and incorporated total imaging matrix coil technology, which allowed for comprehensive body coverage using multiple integrated radiofrequency surface coils. Additionally, the system featured a fully operational PET component with avalanche photodiode technology, which was integrated within a magnetic resonance gantry. The examination protocol involved a comprehensive scan of the entire body, with a specific focus on the thoracic region. To begin, a coronal fast-view T1-weighted MR localizer sequence was conducted to obtain scout images (Table 1). Subsequently, a whole-body PET scan was performed from the head to the upper thigh, covering 4-bed positions. Each bed position had an acquisition time of 4 min. Simultaneously, a whole-body T2-weighted MRI was conducted in the same 4-bed positions. This involved using a sagittal short tau inversion recovery (STIR) sequence and a transverse breath-holding half-Fourier single-shot turbo spin-echo (HASTE) sequence. In addition, whole-body diffusion-weighted imaging (DWI) was acquired with 2 b values (i.e., 50 and 1000 s/mm2) in transverse plane. Subsequently, regional PET and MRI were performed simultaneously. Regional PET was performed with an acquisition time of 10 min, whereas dedicated MRI of the thoracic/esophageal region (from the lower neck to upper abdomen) was performed with T2-weighted BLADE sequence with fat saturation in coronal and axial projections, T1-weighted volumetric interpolated breath-hold examination (VIBE) sequence in transverse plane, and corresponding axial DWI (b = 50, 1000 s/mm2). Following DWI, axial dynamic contrast-enhanced MRI (DCE-MRI) using a three-dimensional (3D) T1-weighted spoiled gradient-echo sequence was obtained by intravenously injecting a standard dose (0.1 mmol/kg body weight) of gadopentetate dimeglumine (Gd-DTPA; Magnevist; Bayer-Schering, Burgess Hill, UK) at a rate of 3 mL/s. The temporal resolution was 6.8 s, with a total acquisition time of 272 s (40 phases). After DCE-MRI, contrast-enhanced MRI using a T1-weighted VIBE sequence with fat saturation was conducted for a dedicated regional scan in axial, coronal, and sagittal projections and a final whole-body scan in transverse plane. The PET data were reconstructed using an ordinary Poisson ordered subset expectation maximization, with three iterations, 21 subsets, and a 4-mm Gaussian post-processing filter, into 344 × 344 matrices.

**Table 1:** MRI sequences parameters used for integrated PET/MRI

	Region	Sequence	TR	TE	ST	FOV	VS	T
Pre contrast	Whole body	COR_T1 FastView	2.56	1.44	5	480	5.0 × 5.0 × 5.0	00:26
	Whole body	TRA_T2 HASTE	1000	84	6	380	0.6 × 0.6 × 6.0	02:24
	Whole body	SAG_T2 STIR	3400	57	4	264	1.0 × 1.0 × 4.0	06:12
	Whole body	TRA_DWI (b = 50, 1000)	14,800	59	5	380	1.6 × 1.6 × 5.0	05:26
	Whole body	TRA_ADC	14,800	59	5	380	1.6 × 1.6 × 5.0	
	Thorax (Esophagus)	COR_T2 BLADE FS	2000	104	5	300	1.2 × 1.2 × 5.0	03:19
	Thorax (Esophagus)	TRA_T2 BLADE FS	2000	83	5	320	0.5 × 0.5 × 5.0	02:24
	Thorax (Esophagus)	TRA_T1 VIBE	4.41	1.95	3	400	0.7 × 0.7 × 3.0	00:18
	Thorax (Esophagus)	TRA_DWI (b = 50, 1000)	14,800	59	5	380	1.6 × 1.6 × 5.0	02:43
	Thorax (Esophagus)	TRA_ADC	14,800	59	5	380	1.6 × 1.6 × 5.0	
Post contrast	Thorax (Esophagus)	TRA_DCE-MRI	3.35	1.17	3	360	1.4 × 1.4 × 3.0	04:32
	Thorax (Esophagus)	TRA_T1 C + VIBE FS	4.41	1.95	3	400	0.7 × 0.7 × 3.0	00:17
	Thorax (Esophagus)	COR_T1 C + VIBE FS	3.23	1.16	3	400	0.6 × 0.6 × 3.0	00:17
	Thorax (Esophagus)	SAG_T1 C + VIBE FS	3.38	1.2	1.5	360	0.6 × 0.6 × 1.5	00:18
	Whole body	TRA_T1 C + VIBE FS	4.41	1.95	3	400	0.7 × 0.7 × 3.0	01:08

TR = repetition time in ms; TE = echo time in ms; ST = Slice thickness in mm; FOV = field of view in mm; VS = voxel size in mm; T = scanning time in min; TRA = transverse; HASTE = half-Fourier single-shot turbo spine echo; SAG = sagittal; STIR = short tau inversion recovery; DWI = diffusion-weighted imaging; ADC = apparent diffusion coefficient; COR = coronal; BLADE (Siemens Healthcare, Erlangen, Germany) = the trade name of a variation of the PROPELLER (Periodically Rotated Overlapping ParallEL Lines with Enhanced Reconstruction) technique; FS = fat saturation; VIBE = volumetric interpolated breath-hold examination; DCE-MRI = dynamic contrast-enhanced MRI

Since MRI scans can produce artifacts that affect imaging interpretation, reducing artifacts is crucial. For DCE-MRI, we optimized temporal resolution and used parallel imaging techniques to lessen scan time and motion artifacts. Saturation bands were placed outside the imaging area to minimize flow artifacts. In DWI, we utilized Echo Planar Imaging (EPI) correction methods to mitigate geometric distortions and employed proper shimming to correct for magnetic field inhomogeneities. Furthermore, we instruct patients on breath-holding and ensure their comfortable position to reduce patient-related artifacts, such as voluntary motion. We also suggest pain relievers for those experiencing CRT-related esophageal discomfort to minimize disruptions.

### Treatment protocol

The disease staging and treatment protocols underwent a thorough review and validation process by the esophageal cancer committee at our institution. Patients were staged according to the 8th American Joint Committee on Cancer (AJCC) staging criteria. The patients received neoadjuvant CRT with surgery or definitive CRT depending on the treatment protocol of our hospital. If the clinical stage was T2N0M0 or above, patients were given the option of receiving neoadjuvant CRT followed by esophagectomy. Patients who were deemed unsuitable for surgery due to significant comorbidities, tumors in the cervical area, or personal refusal of surgery were administered definitive CRT. This study utilized two chemotherapy regimens, namely TC (paclitaxel and carboplatin) and PF (cisplatin and 5-fluorouracil). The TC regimen consisted of a weekly combination of carboplatin and paclitaxel. On the other hand, the PF regimen involved the administration of 5-fluorouracil for four consecutive days, along with cisplatin, repeated every three weeks. Concurrent radiotherapy was administered at a dosage range of 45–60 Gy for definitive CRT and 40–45 Gy for neoadjuvant CRT.

### Post-therapy surveillance

The surveillance protocol consisted of regular follow-up visits at intervals of three months for the initial two years, followed by visits every six months during the third and fourth years, and subsequently every 6–12 months. Additionally, the patients underwent contrast-enhanced CT scans every six months for the first two years, and then annually thereafter. Endoscopy was performed if the patient had symptoms of dysphagia.

### Image analysis

Tumor segmentation in the PET images was performed using the PMOD software package (PMOD Technologies Ltd., Zurich, Switzerland). First, boundaries were drawn by an experienced nuclear medicine physician (blinded to the clinical data), large enough to include the primary tumor in the axial, coronal, and sagittal [18 F]FDG-PET scans. The volumes of interest (VOIs) were carefully examined and confirmed by an experienced nuclear medicine physician. Subsequently, the boundaries of the tumors were determined using 40% of the maximum standardized uptake value (SUV) within the VOI [10]. Finally, the SUV and total lesion glycolysis (TLG) of the lesion were automatically calculated using the software.

All MRI datasets of each patient were meticulously evaluated by an experienced radiologist. With the aid of T2-weighted, T1-weighted post-contrast, and DWI, the relevant images depicting esophageal cancer within the T2-weighted and DCE thoracic/esophageal datasets were identified and selected. Using our in-house software written in MATLAB 7.0 (The Mathworks, Natick, MA, USA), the radiologist manually demarcated the region of interest (ROI) of the tumor within each selected T2-weighted and DCE image. Each ROI was drawn carefully to avoid areas with liquefaction, necrosis, or air-filled pockets to the best extent possible. Those clearly identified esophageal cancers despite artifacts were taken into ROI drawing; however, those poorly visualized cancers because of artifacts were excluded from ROI drawing.

These manually delineated ROIs were then automatically superimposed onto the corresponding apparent diffusion coefficient (ADC) and DCE-derived pharmacokinetic maps by our software. A tumoral histogram is subsequently generated from the ROI on each ADC and pharmacokinetic map. By integrating all individual histograms derived from the ADC and pharmacokinetic maps, our software yielded a comprehensive ADC histogram and various pharmacokinetic colormaps representing the entire esophageal cancer of each patient. The extended Kety model was used in a voxel-wise manner for pharmacokinetic analysis [11]. The arterial input function was extracted using a blind source separation algorithm [12]. The ROIs were manually drawn on the DCE-MRI by the same head and neck radiologist. The following pharmacokinetic parameters were calculated: volume transfer constant (*K*^*trans*^), rate constant (*K*_*ep*_), extravascular extracellular volume fraction (*V*_*e*_), and initial area under the curve (iAUC).

In the course of chemoradiotherapy, changes in the biomarker SUV, TLG, *K*^*trans*^, *K*_*ep*_, *V*_*e*_, or iAUC were calculated as: Δbiomarker = 100 x [biomarker value on the interim PET/MRI – biomarker value on the baseline PET/MRI] / biomarker value on the baseline PET/MRI.

### Clinical response

The clinical response was assessed through an impartial evaluation of contrast-enhanced CT images obtained three months post-treatment, in comparison to the initial scans. Tumor response was determined on CT scans using the Response Evaluation Criteria in Solid Tumors (RECIST) version 1.1, with classifications including complete response (CR), progressive disease (PD), partial response (PR) or stable disease (SD) [13]. Patients who achieved PR, SD, or PD were classified in the non-complete response group.

### Statistical analysis

In this study, the clinical response was used as the benchmark for evaluating treatment response. The efficacy of PET/MRI in predicting treatment outcomes was assessed through the computation of the area under the receiver operating characteristic (ROC) curve. To determine the statistical significance of the predictive power, the 95% confidence intervals (CI) for the area under the curve (AUC) and the significance level (P value) of the test were calculated using bootstrap techniques with 1,000 replicates. The Delong method was utilized to evaluate the statistical significance of the observed area under the curve (AUC) in comparison to a null hypothesis of 0.5. A one-sided P value was calculated to determine the significance of the observed AUC [14]. We examined a range of sequential cut-off points for each imaging biomarker. The threshold corresponding to the lowest p-value was selected as the optimal cut-off point for subsequent analyses. Progression-free survival (PFS) was determined by measuring the time from the date treatment initiates to the occurrence of disease progression or recurrence. Overall survival (OS) was determined by measuring the time from the date of diagnosis to either death from any cause or the last follow-up. The relationship between PET/MRI parameters and survival outcomes was visually represented using Kaplan-Meier product limit curves and evaluated using the log-rank test. Variables showing a p-value significance of < 0.05 following univariate analyses were considered for inclusion in the multivariate Cox regression model using a backward elimination approach. The prognostic model, derived from baseline and interim PET/MRI biomarkers, was established using independent risk factors. Internal validation of the models was carried out through the bootstrapping technique, involving the generation of 1000 bootstrap samples from the original dataset with replacement. The bootstrapping procedure was executed using the R programming language. All statistical analyses were conducted using MedCalc version 19.1.5 and SPSS software version 20. Statistical significance was operationally defined as a two-tailed P value that was found to be less than 0.05.

## Results

### Baseline patient characteristics

Between August 2018 and July 2021, we enrolled 54 patients in this study. We excluded 14 patients from the final analysis: 4 were diagnosed with M1 disease, 6 withdrew from the study and did not complete the interim PET/MRI scan, 1 was lost during follow-up, and 3 had primary tumors that were too small and obscured by prominent artifacts to achieve optimal DCE-MRI or DWI results. Out of the six patients who withdrew from the study and did not complete the interim PET/MRI scan, four patients voluntarily terminated their treatment processes, one patient was transferred to another hospital for ongoing treatment, and another patient was unable to lie on the PET/MRI table due to the underlying medical condition. The information pertaining to the 40 patients with M0 stage cancer, which were considered for the final analysis, is presented in Table 2.

**Table 2:** General characteristics of the study participants

Variable	Number of patients (%)
Age (years), mean ± SD	56 ± 8
Gender	
Male	38 (90)
Female	2 (10)
Tumor site	
Cervical	3 (7)
Upper-third thoracic	8 (20)
Middle-third thoracic	16 (40)
Lower-third thoracic	13 (33)
Overall stage	
II	5 (13)
III	23 (58)
IV	12 (29)
T classification	
T2	7 (18)
T3	23 (58)
T4	10 (24)
N classification	
N0	3 (7)
N1	11 (27)
N2	19 (48)
N3	7 (18)
Treatment	
nCRT + surgery	13 (33)
dCRT	27 (67)

Data are expressed as counts and percentages (in parentheses), unless otherwise indicated. SD = standard deviation;

nCRT = neo-adjuvant chemoradiotherapy; dCRT = definitive chemoradiotherapy

Thirteen patients received neoadjuvant chemoradiotherapy followed by surgery and 27 underwent definitive chemoradiotherapy. The cohort as a whole had a median follow-up time of 38 months, with a range of 8 to 70 months. Fourteen patients died and nineteen patients developed a progressive or recurrent disease at the end of the follow-up period.

### The predictive power of PET/MRI biomarkers for clinical response

**Fig. 1:**
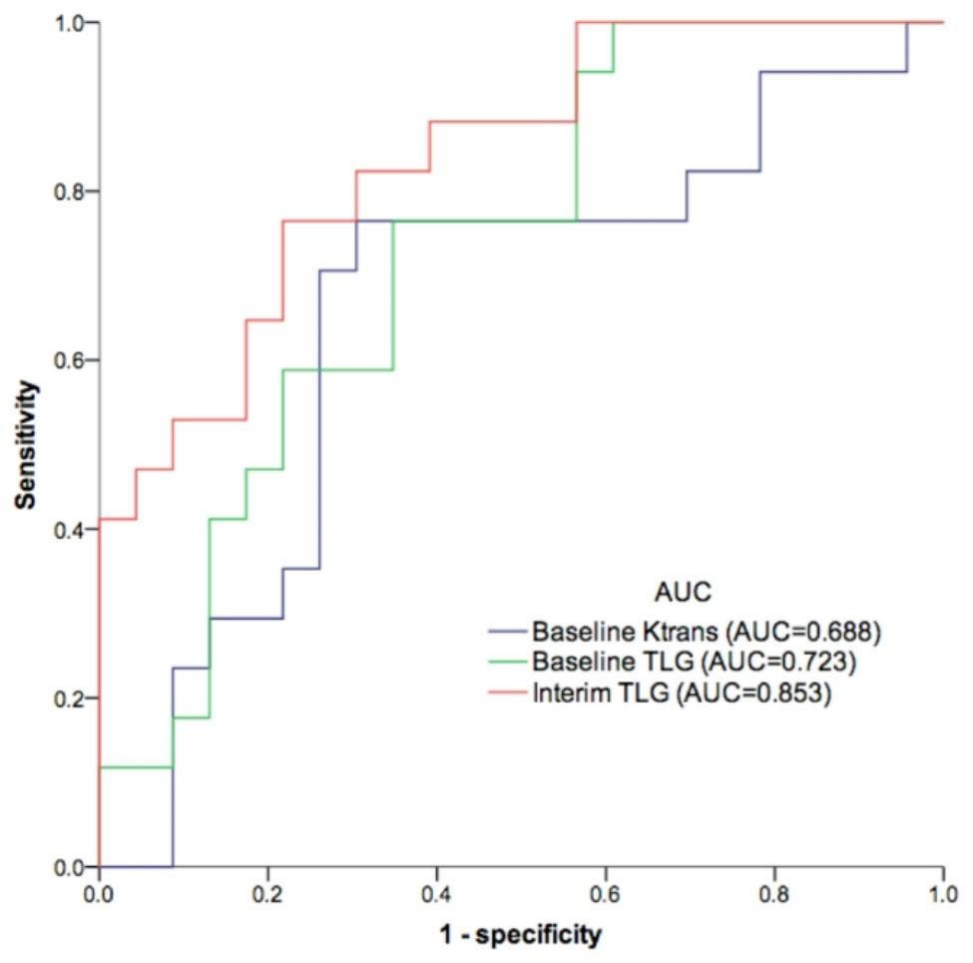
Areas under the receiver operating characteristic curves (AUCs) for baseline TLG, interim TLG, and baseline *K*^*trans*^

After complete treatment, 23 (57.5%) patients achieved complete response (CR group) and the remaining 17 had residual disease (non-CR group). The predictive power of TLG on baseline PET/MRI (Baseline TLG) for clinical response was statistically significant (Supplementary Tables 1, Additional File 1). The predictive power of TLG from the interim PET/MRI (Interim TLG) was also significant. But the predictive capacity of each Δbiomarker on PET/MRI was not significant. Among the PET/MRI parameters, baseline TLG (area under the curve (AUC) = 0.723, *P* = 0.006), or interim TLG (AUC = 0.853, *P* < 0.001), and baseline *K*^*trans*^ (AUC = 0.688, *P* = 0.034) showed moderate-to-high AUCs for predicting clinical response (Fig. 1). The baseline TLGs or interim TLGs in the CR group were significantly lower than those in the non-CR group (*P* = 0.013 and < 0.001, respectively; Table 3). A trend toward a lower *K*^*trans*^ was observed in the CR group.

**Table 3:** Comparison of PET/MRI parameters between the CR and non-CR groups

PET/MRI parameters	CR group median ± IQR	Non-CR group median ± IQR	*P* value
Baseline *K*^trans^	83.960 ± 29.225	138.400 ± 122.100	0.087
Interim *K*^trans^	125.580 ± 68.690	120.510 ± 37.665	0.520
*ΔK* ^trans^	54.774 ± 94.165	-13.377 ± 36.951	0.359
Baseline *K*_ep_	71.330 ± 53.925	104.640 ± 64.240	0.265
Interim *K*_ep_	68.310 ± 54.088	90.560 ± 62.265	0.232
*ΔK*_ep_	-6.824 ± 52.085	-0.846 ± 36.123	0.626
Baseline *V*_e_	192.990 ± 59.943	269.180 ± 87.690	0.315
Interim *V*_e_	232.580 ± 75.310	211.860 ± 56.485	0.607
*ΔV*_e_	3.114 ± 47.243	-5.304 ± 23.312	0.386
Baseline iAUC	415.710 ± 145.535	551.010 ± 123.675	0.705
Interim iAUC	466.790 ± 224.903	642.550 ± 174.445	0.107
ΔiAUC	10.601 ± 74.505	33.293 ± 23.762	0.265
Baseline ADC_mean_	1193.450 ± 610.553	1060.280 ± 510.645	0.665
Interim ADC_mean_	1251.010 ± 606.508	1072.120 ± 553.610	0.551
ΔADC_mean_	-0.229 ± 14.129	5.828 ± 6.820	0.978
Baseline SUVmax	15.150 ± 2.638	18.500 ± 3.340	0.062
Interim SUVmax	8.390 ± 2.620	10.620 ± 1.720	0.042
ΔSUVmax	-48.636 ± 11.937	-36.307 ± 10.398	0.302
Baseline TLG	146.230 ± 91.748	290.430 ± 72.225	0.013
Interim TLG	30.820 ± 25.108	98.910 ± 38.750	< 0.001
ΔTLG	-71.546 ± 16.773	-53.436 ± 16.810	0.464

IQR = interquartile range; CR = complete response; SUV_max_ = maximum standardized uptake value; TLG = total lesion glycolysis; ADC_mean_ = mean apparent diffusion coefficient; *K*^*trans*^ = volume transfer constant; *K*_ep_ = flux rate constant; *V*_e_ = extracellular volume ratio; iAUC = initial area under curve

*P* value, comparison of the parameters between CR group and non-CR group

### PET/MRI biomarkers in predicting progression-free and overall survival

**Fig. 2:**
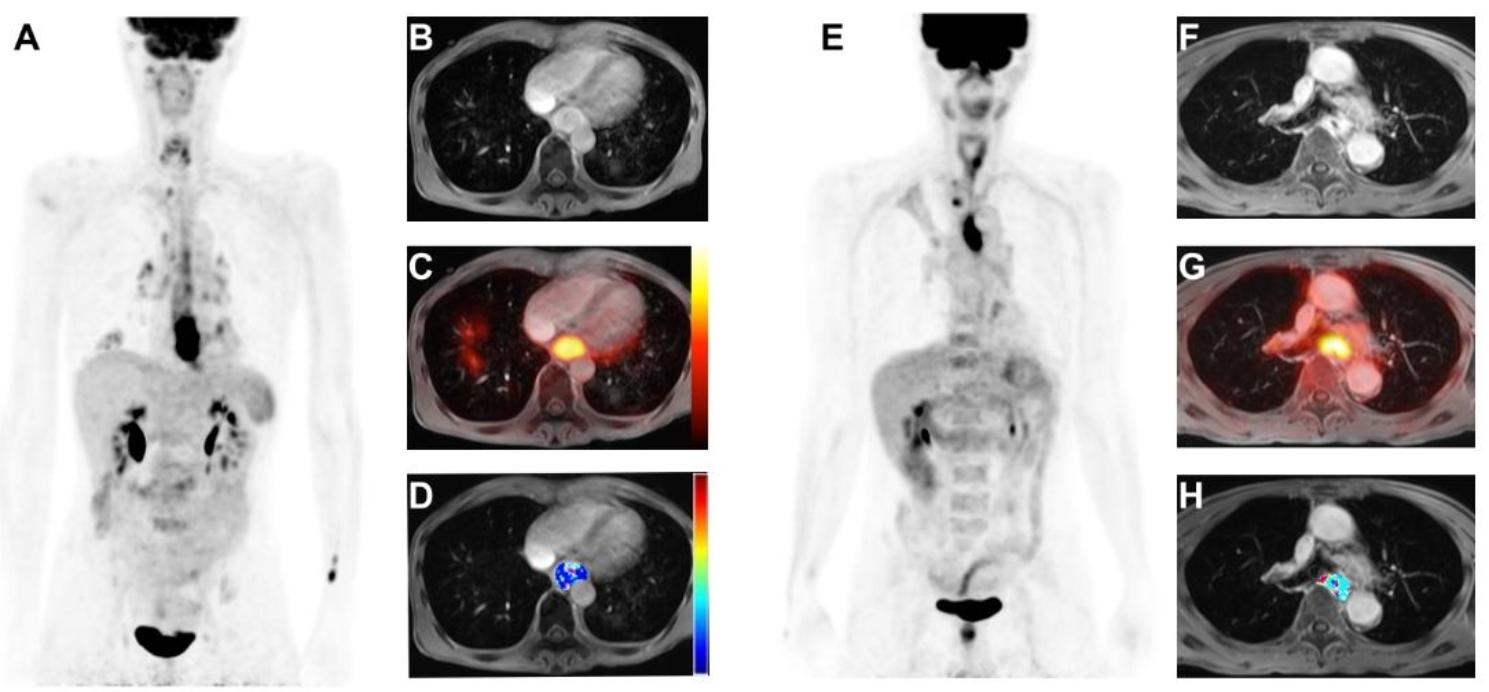
A combination of PET and MRI functional biomarkers in predicting overall survival. (**A–D**) Representative axial images of a lower-third esophageal cancer patient with T3N2 disease. (**A**) Interim maximal intensity projection PET image; (**B**) Interim contrast-enhanced MR image; (**C**) Fused interim [18F]FDG-PET/MRI image; and (**D**) Interim contrast-enhanced MR image with an overlaid *Ve* map of the primary tumor. This patient had an interim TLG of 113 and an interim *Ve* value of 34. He died of tumor recurrence with a short overall survival period of 8 months after definitive chemoradiotherapy. (**E–H**) Images of an upper-third esophageal carcinoma patient with T4bN2 disease. (**E**) Interim maximal intensity projection PET image; (**F**) Interim contrast-enhanced MR image; (**G**) Fused interim [18F]FDG-PET/MRI image; and (**H**) Interim contrast-enhanced MR image with an overlaid *Ve* map of the primary tumor. He had an interim TLG of 45 and a *Ve* value of 162. This patient still survived without disease recurrence for four years after definitive chemoradiotherapy

The univariate analysis identified the following variables as significant risk factors for OS: interim SUVmax, interim TLG, interim *Ve*, and SUVmax change between the baseline and interim scans (ΔSUVmax) (Table 4). Baseline SUVmax, baseline TLG, baseline *K*^*trans*^, baseline *Ve*, interim SUVmax, interim TLG, ΔSUVmax, Δ*K*^*trans*^, and treatment were significantly associated with PFS. Multivariate analysis showed that interim *V*_*e*_ (*P* = 0.001), and interim TLG (*P* < 0.001) retained their independent prognostic significance for OS. High baseline *V*_*e*_ (*P* = 0.044) and high interim TLG (*P* = 0.004) remained adverse prognostic factors for PFS. Figure 2 displays representative cases to illustrate the associations of PET and MR functional markers from interim PET/MRI with survival outcomes.

**Table 4:** Univariate analysis of clinical factors and PET/MRI biological imaging markers in relation to overall survival and progression-free survival in patients with esophageal squamous cell carcinoma

Variable	OS		PFS	
	Patient no. (event no.)	*P* value	Patient no. (event no.)	*P* value
Age (years)		0.576		0.201
≤ 56	21 (7)		21 (12)	
> 56	19 (7)		19 (7)	
Sex		0.170		0.423
Male	38 (12)		38 (17)	
Female	2 (2)		2 (2)	
Tumor site		0.710		0.969
Cervical	3 (2)		3 (1)	
Upper-third thoracic	9 (4)		9 (5)	
Middle-third thoracic	17 (5)		17 (8)	
Lower-third thoracic	11 (3)		11 (5)	
Tumor stage		0.368		0.375
I-II	5 (1)		5 (1)	
III-IV	35 (13)		35 (18)	
T classification		0.189		0.346
T1-2	7 (1)		7 (2)	
T3-4	33 (13)		33 (17)	
N classification		0.516		0.770
N0-1	14 (4)		14 (7)	
N2-3	26 (10)		26 (12)	
Treatment		0.144		0.025
nCRT + sugery	13 (3)		13 (3)	
dCRT	27 (11)		27 (16)	
*Imaging Biomarker*				
Baseline SUVmax		0.523		0.027
≤ 20	32 (12)		32 (13)	
> 20	8 (2)		8 (6)	
Baseline TLG (g/mL × mL)		0.246		0.036
≤ 221.8	24 (8)		24 (8)	
> 221.8	16 (6)		16 (11)	
Baseline *K*^*trans*^ (10^− 3^ min^− 1^)		0.306		0.008
≤ 85.5	21 (7)		21 (6)	
> 85.5	19 (7)		19 (13)	
Baseline *K*_*ep*_ (10^− 3^ min^− 1^)		0.324		0.166
≤ 47	13 (4)		13 (4)	
> 47	27 (10)		27 (15)	
Baseline *V*_*e*_ (10^− 3^)		0.088		0.004
≤ 202	31 (9)		31 (12)	
> 202	9 (5)		9 (7)	
Baseline iAUC		0.190		0.921
≤ 235	9 (5)		9 (4)	
> 235	31 (9)		31 (15)	
Baseline ADC_mean_ (10^− 3^ mm^2^/s)		0.900		0.865
≤ 864	16 (4)		16 (8)	
> 864	24 (10)		24 (11)	
Interim SUVmax		0.004		0.027
≤ 8.4	16 (2)		16 (4)	
> 8.4	24 (12)		24 (15)	
Interim TLG (g/mL × mL)		< 0.001		< 0.001
≤ 98.9	29 (6)		29 (8)	
> 98.9	11 (8)		11 (11)	
Interim *K*^*trans*^ (10^− 3^ min^− 1^)		0.147		0.940
≤ 120.5	21 (10)		21 (10)	
> 120.5	19 (4)		19 (9)	
Interim *K*_*ep*_ (10^− 3^ min^− 1^)		0.304		0.426
≤ 159	35 (12)		35 (16)	
> 159	5 (2)		5 (3)	
Interim *V*_*e*_ (10^− 3^)		0.015		0.455
≤ 89	9 (6)		9 (5)	
> 89	31 (8)		31 (14)	
Interim iAUC		0.434		0.631
≤ 235	9 (4)		9 (5)	
> 235	31(10)		31 (14)	
Interim ADC_mean_ (10^− 3^ mm^2^/s)		0.105		0.568
≤ 1251	21 (9)		21 (11)	
> 1251	19 (5)		19 (8)	
Δ SUVmax		0.006		0.047
≤ -48.6 (-32)	16 (2)		30 (12)	
> -48.6 (-32)	24 (12)		10 (7)	
Δ TLG (g/mL × mL)		0.057		0.435
≤ -47.9	27 (7)		27 (12)	
> -47.9	13 (7)		13 (7)	
Δ *K*^*trans*^ (10^− 3^ min^− 1^)		0.235		0.046
≤ 21.2	21 (9)		21 (13)	
> 21.2	19 (5)		19 (6)	
Δ *K*_*ep*_ (10^− 3^ min^− 1^)		0.591		0.082
≤ 8.6	23 (9)		23 (14)	
> 8.6	17 (5)		17 (5)	
Δ *V*_*e*_ (10^− 3^)		0.062		0.116
≤ -44	10 (6)		10 (6)	
> -44	30 (8)		30 (13)	
Δ iAUC		0.372		0.367
≤ 1.7	17 (5)		17 (7)	
> 1.7	23 (9)		23 (12)	
Δ ADC_mean_ (10^− 3^ mm^2^/s)		0.217		0.752
≤ 6.3	24 (10)		24 (10)	
> 6.3	16 (4)		16 (9)	

OS = overall survival; PFS = progression-free survival; TLG = total lesion glycolysis; SUV_max_ = maximum standardized uptake value; ADC_mean_ = mean apparent diffusion coefficient; K^trans^ = volume transfer constant; K_ep_ = flux rate constant; V_e_ = extracellular volume ratio; iAUC = initial area under curve; nCRT = neo-adjuvant chemoradiotherapy; dCRT = definitive chemoradiotherapy

### Prognostic model based on baseline and interim PET/MRI biomarkers

The predictive models were further investigated by summing up the independent PET/MRI biomarkers identified in the multivariate analysis. Prognostic factors were assigned a value of 1 or 0 if present or absent, respectively. Table 5 shows the performance of the prognostic models based on baseline and interim PET/MRI biomarkers.

**Table 5:** Comparison of Harrell’s concordance index between TNM stage and prognostic models based on PET/MRI biomarkers

	OS		PFS	
	c-index	95% CI	c-index	95% CI
TNM stage	0.56	0.49–0.62	0.53	0.45–0.61
PET/MRI model for OS	0.79*	0.66–0.91		
PET/MRI model for PFS			0.70**	0.60–0.80

*P = 0.002 in comparison with TNM stage

** *P* = 0.047 in comparison with TNM stage

CI = confidence interval; OS = overall survival; PFS = progression-free survival

The c-indices of the models incorporating interim TLG with *Ve* in predicting OS and incorporating baseline *Ve* and interim TLG in predicting PFS were 0.79 and 0.68, respectively. These values were significantly higher than the corresponding c-indices of the TNM staging system (*P* = 0.002 and *P* = 0.047, respectively). Upon internal validation through bootstrapping, the c-indices of the PET/MRI prognostic models, based on both baseline and interim biomarkers, exhibited similarity to the values observed in the training cohort (Supplementary Tables 2, Additional File 2).

## Discussion

[18F]FDG PET/MRI has been advocated as a promising tool for the early assessment of treatment outcomes in patients with cancer. However, there is a paucity of studies addressing the utility of baseline and interim PET/MRI in ESCC. This study found that baseline *K*^*trans*^, baseline TLG, and interim TLG had moderate to high AUCs in predicting clinical response to chemoradiotherapy. The interim *Ve* and TLG levels were identified as independent prognostic factors for OS. Moreover, the baseline *Ve* and interim TLG were independent predictors of PFS. The combination of PET metabolic and MRI perfusion parameters from baseline or interim PET/MRI scans aids in predicting the survival of patients with ESCC.

The development of a diagnostic test for early prognostication of survival in patients diagnosed with ESCC holds significant importance. However, no robust molecular markers are currently available to predict the prognosis of these patients. In this study, several PET/MRI parameters showed the potential to predict long-term OS and PFS. Univariate analysis revealed that interim SUVmax, interim TLG, interim *Ve*, and ΔSUVmax were significant predictors of OS. The SUVmax, TLG, *K*^*trans*^, and *Ve* from baseline PET/MRI and SUVmax and TLG from interim PET/MRI were significant prognostic factors for PFS. In multivariate analysis, interim TLG, baseline *Ve*, and interim *Ve* were independent prognostic factors for OS or PFS. Few researchers have addressed the prognostic value of PET/MRI in esophageal cancer. Yu et al. [10] evaluated the prognostic value of [18F]FDG-PET/MRI and found that the MTV/ADC ratio was an independent risk factor in patients with esophageal cancer. However, 30% of their study participants had distant metastases. Given the diverse prognoses of patients with esophageal cancer with or without distant metastasis, the report by Yu et al. cannot be fully applied to patients with M0 esophageal cancer. In our study, only M0 patients were included in the analysis, which may explain the discrepancies between our results and those of Yu et al. In another study, Belmounhand et al. assessed the value of PET/MRI in predicting the response of neoadjuvant chemotherapy in patients with esophageal adenocarcinoma [15]. The results showed that changes in ADC and SUV values could predict the resectability of the esophageal tumor. However, they did not include DCE-MRI in the study protocol. In this study, both DCE-MRI and DWI techniques were used in the PET/MRI examination, which could provide more comprehensive information for clinicians and patients. This will allow for more personalized treatment strategies.

Previous reports have shown that *Ve* is associated with prognosis and treatment outcomes in some cancers [16–18]. The *Ve* parameter reflects the extravascular extracellular space of the tumor. In the study of Wong et al., the relevance of multimodality imaging parameters in patients with head and neck cancer treated with chemoradiation was evaluated [17]. They found that patients with a good response to chemoradiotherapy had a higher *Ve* value in the interim DCE-MRI scan. In this cohort with ESCC, we found that a larger interim *Ve* was associated with a higher OS rate. Besides, patients achieving complete response also had a higher interim *Ve* value, although the difference was not statistically significant. The potential value of the interim PET parameter in predicting survival in esophageal cancer has been evaluated by some investigators. In one study, Li et al. evaluated patients with esophageal cancer treated with definitive chemoradiotherapy [19]. Cox regression analyses revealed interim TLG as an independent prognostic factor for overall survival. Our study also showed that the interim TLG was an independent risk factor for OS, which is comparable to the results of Li et al.’s study. The PET/MRI scan enables the concurrent acquisition of MRI perfusion and PET metabolic indices, thereby offering the clinician a more objective reference.

Patients who do not respond to chemoradiotherapy may experience toxic side effects and delays in receiving effective therapy, significantly affecting their quality of life and survival. Early response assessment to chemoradiotherapy facilitates adaptive changes. In this study, the metabolic parameter TLG from PET/MRI showed acceptable efficacy in predicting chemoradiotherapy response in ESCC patients. A higher baseline TLG level was associated with a poor response to chemoradiotherapy. In a prospective assessment of patients with esophageal cancer treated with chemoradiotherapy, TLG from baseline PET/CT showed a good predictive value for treatment response to chemoradiotherapy [20]. Our results based on PET/MRI scans confirm the significance of baseline TLG in predicting the response in patients with ESCC. An interim scan could identify non-responders who could benefit the most from dose-intensification protocols or those who could be treated with immediate surgery in patients treated with chemoradiotherapy. Previous studies have also shown that interim PET parameters have the potential to predict treatment response after chemoradiotherapy in esophageal cancer [21, 22], but some controversy still exists [7]. Our study showed that the interim TLG predicted the treatment response with a high AUC (0.853), supporting the use of interim PET in these patients.

The perfusion parameter *K*^*trans*^ showed a moderate AUC in predicting clinical response in this study. *K*^*trans*^ is a pharmacokinetic parameter that reflects the rate at which contrast agent is exchanged between the blood plasma and the extravascular extracellular space in a tissue. The predictive value of the MRI perfusion parameter may vary among different malignancies, and this point will require clarification in future research.

In this clinical trial, we have adopted the RECIST criteria to define clinical response, facilitating comparisons with outcomes from other clinical trials. Our multidisciplinary team of experts reviewed and discussed the CT findings for each patient, ultimately reaching a consensus. Currently, the accuracy of CT scans in assessing the TN status of esophageal cancer is suboptimal. As a result, the NCCN guideline recommends considering supplementary techniques, such as endoscopy or [18F]FDG-PET apart from CT scans. Nevertheless, the interpretation of [18F]FDG-PET or endoscopy images tends to be subjective. Different physicians may interpret the results differently, making it less suitable as a standard for evaluating efficacy in cancer clinical trials.

The current study had certain limitations. One limitation was the relatively small size of the study population, which affected the strength of our results. Although there are no significant differences in demographics or TNM staging between patients who underwent neoadjuvant CRT with surgery and those who received definitive CRT (Supplementary Tables 3, Additional File 3), future prospective studies with a large sample size are needed to confirm the results of this study. Second, our study included patients who underwent neoadjuvant chemoradiotherapy with surgery or definitive chemoradiotherapy. Due to the small number of enrolled patients, we did not analyze the two groups separately based on statistical power considerations. Despite these limitations, our data have implications for patients with esophageal cancer. Our findings contribute to the existing body of literature, particularly in light of the growing utilization of interim PET/MRI. Moreover, our results provide a foundation for future clinical trials.

## Conclusions

Combining baseline and interim [18F]FDG-PET/MRI qualitative imaging parameters provides complementary information, resulting in a higher predictive value in esophageal squamous cell carcinoma. [18F]FDG-PET/MRI may serve as a single-step imaging modality to acquire PET metabolic and MRI perfusion prognostic indices. Further prospective studies are needed to validate the results of this preliminary study.

### Electronic supplementary material

Below is the link to the electronic supplementary material.

Additional File 1: Supplementary Table 1. Predictive power of PET/MRI parameters for clinical response in patients with ESCC.

Additional File 2: Supplementary Table 2. Prognostic performance of TNM stage and PET/MRI prognostic models in the training and validation cohorts.

Additional File 3: Supplementary Table 3. Comparison of demographic data between patients who received nCRT with surgery vs dCRT.

## Data Availability

The datasets used and/or analyzed in this study are available from the corresponding author upon reasonable request.

## Abbreviations

M0: Without distant metastasis
ESCC: Esophageal squamous cell carcinoma
[18F]FDG: ^18^ F-fluorodeoxyglucose
PET/MRI: Positron emission tomography–magnetic resonance imaging
CRT: Chemoradiotherapy
DWI: Diffusion-weighted MR imaging
DCE-MRI: Dynamic contrast-enhanced MRI
HASTE: Half-Fourier single-shot turbo spin-echo
STIR: Short tau inversion recovery
VIBE: Volumetric interpolated breath-hold examination
OS: Overall survival
PFS: Progression-free survival
TLG: Total lesion glycolysis
SUV: Standardized uptake value
*K*^*trans*^: Volume transfer constant
*K*_*ep*_: Rate constant
AUC: Area under the curve
*Ve*: Extracellular volume fraction
RECIST: Response Evaluation Criteria in Solid Tumors
