# Supplementary material for: Baseline and interim [18F]FDG-PET/MRI to assess treatment response and survival in patients with M0 esophageal squamous cell carcinoma treated by curative-intent therapy
Source: Cancer Imaging. 2023 Nov 6;23:109. doi: 10.1186/s40644-023-00630-2 (PMC10629192; doi:10.1186/s40644-023-00630-2)
Supplement: Supplementary file 1 — Additional File 1: Supplementary Table 1. Predictive power of PET/MRI parameters for clinical response in patients with ESCC. [file 40644_2023_630_MOESM1_ESM.docx]

| **Supplementary Table 1** Predictive power of PET/MRI parameters for clinical response in patients with ESCC. | | |
| --- | --- | --- |
|  |  |  |
| Parameter | AUC | *P* value |
|  |  |  |
| Baseline ADCmean | 0.454 | 0.623 |
| Baseline K^trans^ | 0.688 | 0.034 |
| Baseline k_ep_ | 0.583 | 0.372 |
| Baseline V_e_ | 0.611 | 0.259 |
| Baseline iAUC | 0.526 | 0.788 |
| Baseline SUV_max_ | 0.659 | 0.076 |
| Baseline TLG | 0.723 | 0.006 |
| Interim ADC_mean_ | 0.438 | 0.500 |
| Interim K^trans^ | 0.533 | 0.732 |
| Interim k_ep_ | 0.59 | 0.327 |
| Interim V_e_ | 0.457 | 0.651 |
| Interim iAUC | 0.647 | 0.106 |
| Interim SUV_max_ | 0.707 | 0.012 |
| Interim TLG | 0.853 | <0.001 |
| ΔADC | 0.495 | 0.955 |
| ΔK^trans^ | 0.413 | 0.353 |
| Δk_ep_ | 0.466 | 0.727 |
| ΔV_e_ | 0.408 | 0.326 |
| ΔiAUC | 0.609 | 0.238 |
| ΔSUV_max_ | 0.625 | 0.181 |
| ΔTLG | 0.63 | 0.147 |
| TLG = total lesion glycolysis; SUV_max_ = maximum standardized uptake value; ADC_mean_ = mean apparent diffusion coefficient; K^trans^ = volume transfer constant; k_ep_ = flux rate constant; V_e_ = extracellular volume ratio; iAUC = initial area under curve | | |
