# Supplementary material for: Baseline and interim [18F]FDG-PET/MRI to assess treatment response and survival in patients with M0 esophageal squamous cell carcinoma treated by curative-intent therapy
Source: Cancer Imaging. 2023 Nov 6;23:109. doi: 10.1186/s40644-023-00630-2 (PMC10629192; doi:10.1186/s40644-023-00630-2)
Supplement: Supplementary file 2 — Additional File 2: Supplementary Table 2. Prognostic performance of TNM stage and PET/MRI prognostic models in the training and validation cohorts. [file 40644_2023_630_MOESM2_ESM.docx]

| **Supplementary Table 2** Prognostic performance of TNM stage and PET/MRI prognostic models in the training and validation cohorts | | | | | | | | | |
| --- | --- | --- | --- | --- | --- | --- | --- | --- | --- |
|  | **Training cohort** | | | |  | **Validation cohort** | | | |
|  | **Overall survival** | | **Progression-free survival** | |  | **Overall survival** | | **Progression-free survival** | |
|  | **c-index** | **95% CI** | **c-index** | **95% CI** |  | **c-index** | **95% CI** | **c-index** | **95% CI** |
| TNM stage | 0.56 | 0.49-0.62 | 0.53 | 0.45-0.61 |  | 0.56 | 0.48-0.63 | 0.54 | 0.50-0.61 |
| PET/MRI prognostic model for OS | 0.79 | 0.66-0.91 | - | - |  | 0.79 | 0.66-0.90 | - | - |
| PET/MRI prognostic model for PFS | - | - | 0.70 | 0.60-0.80 |  | - | - | 0.71 | 0.59-0.80 |
| CI = confidence interval; OS = overall survival; PFS = progression-free survival. | | | | | | | | | |
