# Supplementary material for: Baseline and interim [18F]FDG-PET/MRI to assess treatment response and survival in patients with M0 esophageal squamous cell carcinoma treated by curative-intent therapy
Source: Cancer Imaging. 2023 Nov 6;23:109. doi: 10.1186/s40644-023-00630-2 (PMC10629192; doi:10.1186/s40644-023-00630-2)
Supplement: Supplementary file 3 — Additional File 3: Supplementary Table 3. Comparison of demographic data between patients who received nCRT with surgery vs dCRT. [file 40644_2023_630_MOESM3_ESM.docx]

| **Supplementary Table 3 Comparison of demographic data between patients who received nCRT with surgery and those underwent dCRT** | | | |
| --- | --- | --- | --- |
| **Variable** | **nCRT+surgery** | **dCRT** | **P value** |
| Age (years), mean | 58 | 56 | 0.529 |
| Gender |  |  |  |
| Male | 13 | 25 | 0.320 |
| Female | 0 | 2 |  |
| Tumor site |  |  | 0.293 |
| Cervical | 0 | 3 |  |
| Upper-third thoracic | 1 | 8 |  |
| Middle-third thoracic | 10 | 7 |  |
| Lower-third thoracic | 2 | 9 |  |
| Overall stage |  |  | 0.529 |
| II | 1 | 4 |  |
| III | 7 | 13 |  |
| IV | 5 | 10 |  |
| T classification |  |  | 0.809 |
| T2 | 2 | 5 |  |
| T3 | 9 | 14 |  |
| T4 | 2 | 8 |  |
| N classification |  |  | 0.279 |
| N0 | 0 | 3 |  |
| N1 | 3 | 8 |  |
| N2 | 6 | 13 |  |
| N3 | 4 | 3 |  |
| nCRT = neo-adjuvant chemoradiotherapy; dCRT = definitive chemoradiotherapy. | | | |
